# Ultraviolet light‐C increases antioxidant capacity of the strawberry (*Fragaria x ananassa*) in vitro and in high‐fat diet‐induced obese rats

**DOI:** 10.1002/fsn3.487

**Published:** 2017-06-20

**Authors:** Cecilia I. Oviedo‐Solís, Cuauhtémoc Sandoval‐Salazar, Edmundo Lozoya‐Gloria, Genaro A. Maldonado‐Aguilera, Herlinda Aguilar‐Zavala, Vicente Beltrán‐Campos, Victoriano Pérez‐Vázquez, Joel Ramírez‐Emiliano

**Affiliations:** ^1^ Departamento de Medicina y Nutrición Universidad de Guanajuato León Gto. México; ^2^ División de Ciencias de Salud e Ingenierías Departamento de Enfermería y Obstetricia Universidad de Guanajuato Celaya Gto. México; ^3^ Laboratorio de Bioquímica y Biología Molecular de Productos Naturales de Plantas CINVESTAV Irapuato, Gto México; ^4^ División de Ciencias de Salud e Ingenierías Departamento de Enfermería Clínica Universidad de Guanajuato Celaya Gto. México; ^5^ División de Ciencias de la Salud Departamento de Ciencias Médicas Campus León Universidad de Guanajuato León México

**Keywords:** high‐fat diet‐induced obese rats, obesity, strawberry antioxidant capacity, ultraviolet light‐C

## Abstract

Flavonoids and polyphenols from the strawberry and other fruits have been proposed to reduce the oxidative stress produced by the obesity and her complications. Moreover, it has been proposed that irradiation with UV‐C to strawberry may increase the antioxidant capacity of this fruit. The aim of the present study was to explore the effects of the UV‐C on antioxidant capacity of strawberry in vitro and in vivo. Strawberry slices were irradiated with ultraviolet light‐C (UV‐C) at 1.2 W/m^2^/16.5 min; then, the power antioxidant was isolated from the nonirradiated and irradiated strawberry slices into an organic phase, which was lyophilized to finally producing a nonirradiated strawberry extract (NSE) and UV‐irradiated strawberry extract (UViSE) powder. After the antioxidant capacity of both extracts were determined in vitro using the Trolox equivalent antioxidant capacity (TEAC) assay and in vivo using high‐fat diet‐induced obese rats. Our results demonstrated that irradiation with UV‐C to strawberry slices increased the antioxidants content, which was corroborated in vitro, where the antioxidant capacity of UViSE was higher than the NSE. However, in obese rats, the reduction in the oxidative damage by the UViSE and NSE were similar in peripheral tissues. Interestingly, the UViSE was better than the NSE to reduce the oxidative damage in brain. In conclusion, UV‐irradiation increases the antioxidants content of strawberry that is correlated with an increased antioxidant capacity in vitro, but in rats, this antioxidant capacity may be more effective in brain than in peripheral tissues.

## 1 INTRODUCTION

Sedentary lifestyle and unhealthy food habits are considered the main culprits of obesity (Han, Lawlor, & Kimm, 2010). Moreover, hypercaloric diets that contain large amounts of refined sugars and fat produce brain damage and systemic oxidative stress (Freeman, Haley‐Zitlin, Rosenberger, & Granholm, 2014). For instance, high‐fat diet (HFD) stimulates the accumulation of adipose tissue, leading to the development of obesity, which is associated with increased oxidative stress in both humans and animal models (Hunsche et al., 2017; Panchal et al., 2011; Salmon, 2016). Thus, the oxidative stress produces a cellular dysregulation, increased production of proinflammatory molecules, energy imbalance, and increases the risk factors for type 2 diabetes, hypertension, hyperlipidemia, and brain damage (Ye, Zhang, Townsend, & Tew, 2015). Therefore, it is necessary to propose strategies to enhance antioxidant defenses and to decrease oxidative stress, hence preventing the development of the obesity′s complications.

The strawberries have phytochemicals with potent antioxidant and anti‐inflammatory activity, like anthocyanins, ellagic acid, caffeic acid, ellagic acid, and certain flavonoids including anthocyanins, tannins, catechin, quercetin, kaempferol, gallic acid derivatives, and have also vitamins C, E and carotenoids (Giampieri, Alvarez‐Suarez, & Battino, 2014; Giampieri et al., 2014; Kårlund et al., 2014). It has been demonstrated that dietary supplementation with the antioxidant curcumin reduces oxidative stress (Martínez‐Morúa et al., 2013), and reduces the brain damage by increasing the brain derived neurotrophic factor (BDNF) levels in obesity and diabetes mice (Franco‐Robles et al., 2014). Interestingly, the berry diet increases the expression of the neuroprotective trophic factor (IGF‐1) in rat brain, suggesting that berries are potent regulators of brain signaling that is correlated to enhancement in cognitive function (Shukitt‐Hale et al., 2008). In experimental animal models fed a high‐fat diet, the strawberry was shown to reduce obesity and improve glycemic control (Prior et al., 2008). In rats, ^56^Fe caused neurochemical changes in frontal cortex and hippocampus, and increased the inflammation and oxidative stress; whereas the strawberry diet significantly reduced neurotoxicity produced by the irradiation with ^56^Fe (Poulosea, Bielinskia, Carrihill‐Knollb, Rabinb, & Shukitt‐Hale, 2014).

Similarly it has been described that the strawberry extracts are scavengers of free radicals (Basu et al., 2009), and the ellagic acid isolated from the strawberry prevents the oxidation of low density lipoprotein (LDL) inducing proliferation of rat aortic smooth muscle cells (Chang, Yu, Chiang, & Tseng, 2008). Moreover, supplementation of freeze‐dried strawberry powder to women with metabolic syndrome decreased the seric levels of lipid peroxidation and cholesterol (Basu et al., 2009). Thus, strawberry flavonoids play a beneficial role in human health.

On the other hand, the irradiation with ultraviolet light (UV) increased the antioxidant capacity by increasing the polyphenols and flavonoids content in fresh‐cut fruits (Alothman, Bhat, & Karim, 2009; Younis, Hasaneen, & Abdel‐Aziz, 2010); however, high dose of UV can produce oxidation of the bioactive compounds (Rivera‐Pastrana, Gardea, Yahia, Martínez‐Téllez, & González‐Aguilar, 2014). Therefore, the effectiveness of UV radiation depends of factors such as dose, light source, species, cultivar, etc. (Ayala Gil & Lozoya Gloria, 2016). With respect to UV radiation, Ayala and others described that this irradiation increases the concentration of phenolic compounds and various antioxidants in strawberries (Ayala Gil & Lozoya Gloria, 2016). Thus, in the present study, the aim was to produce extract of UV‐irradiated strawberry and to determine his effect on the oxidative stress in HFD‐fed rats.

## 2 MATERIAL AND METHODS

### 2.1 Production of strawberry extract

Strawberry extract (SE) was produced by Ayala Gil and others as described previously (Ayala Gil & Lozoya Gloria, 2016). Briefly, strawberries (*Fragaria x ananassa*) were purchased from local producers at Irapuato, Gto., México. The ripe strawberries were cleaned, disinfected, and sliced (6 mm of thick); then these slices were irradiated with ultraviolet light‐C (UV‐C) at 1.2 W/m^2^/16.5 min, and others strawberries slices were used without irradiation. The UV‐irradiated and non‐UV‐irradiated slices were frozen at −20°C followed by lyophilization during 7 days.

To extract the power antioxidant, lyophilized slices were ground. Then, 1 g of the strawberry powders were mixed with 20 ml of methanol:acetic acid (100:1 v/v) for 2 hr at 5°C, following by centrifugation at 500 rpm for 30 min. The supernatant was concentrated in a rotary evaporator (BÜCHI 461) at 39°C; whereas the pellet was mixed with 20 mL of acetone:acetic acid (100:1 v/v) for 2 hr at 5°C and then it also was concentrated in a rotary evaporator at 39°C. The samples concentrated from methanol and acetone were mixed and dissolved together in distilled water. The UV‐irradiated and non‐UV‐irradiated slices were processed separately.

The UV‐irradiated and non‐UV‐irradiated aqueous extracts were incubated at a ratio of 2:1 with 2N HCl in water bath at 100°C for 1 hr. The extracts were cooled and centrifuged at 1200 g for 30 min. The supernatants were incubated at a ratio of 1:1 with ethyl acetate during 2 min; then, the organic phase was recovered; this extraction procedure with ethyl acetate was repeated eight times. Finally, these UV‐irradiated and non‐UV‐irradiated organic extracts were concentrated in a rotary evaporator at 39°C, following by mixed with starch for better handling of these.

### 2.2 Measurement of the anthocyanins content in strawberry extracts

The anthocyanin content was determined as previously described using pelargonidin as standard (Cheng & Breen, 1991).

### 2.3 Measurement of the polyphenols content in strawberry extracts

The polyphenols content in extracts was measured as previously described using galic acid as standard (Slinkard & Singleton, 1977).

### 2.4 Measurement of the flavonoids content in strawberry extracts

The flavonoids content was quantified using a high‐performance liquid chromatography (HPLC) equipped with UV‐Vis Detector (Agilent Technologies 1290 infinity). Specifically, fisetin was determined using a chromatographic column (Zorbax Eclipse Plus C18, 1.8 μm 2.1 × 50 mm) and a mobile phase (water‐acetic acid (92:2, v/v), water‐acetonitrile‐acetic acid (68:30:2, v/v/v)) at 360 nm and 35°C as was described (Fang, Li, Pan, & Huang, 2007). Pelargonidin was determined using a chromatographic column (Hypersil Gold, 1.9 μm 2.1 × 50 mm) and a mobile phase (water‐acetic acid (92:2, v/v), water‐acetonitrile‐acetic acid (110:50:40, v/v/v)) at 510 nm and 30°C as was described (Fazeelat, Afzal, Asif, Zamir, & Saleem, 2007).

### 2.5 Determination of the antioxidant capacity of strawberry extracts

The antioxidant capacity of the nonirradiated strawberry extract (NSE) and UV‐irradiated strawberry extract (UViSE) were determined using the Trolox equivalent antioxidant capacity (TEAC) assay as described by Brand‐Williams and others (Brand‐Williams, Cuvelier, & Berset, 1995). The total antioxidant capacity is expressed as mmol/L of Trolox equivalent (TE)/100 g of dry weight.

### 2.6 Animal care and strawberry extract treatment

Twenty male Wistar rats of 1 month old (100–150 g of weight) were maintained in polypropylene animal cages in a temperature‐controlled environment (22 ± 2°C) and under a light–dark cycle set at 12:12 hr in the University of Guanajuato Animal Facility. All animal procedures were conducted in accordance with the Guide for the Care and Use of Laboratory Animals (Institute of Laboratory Animal Resources 1996) and the Mexican legislations (NOM‐062‐ZOO‐1999).

The rats were randomized into four groups. The first group consisted of five rats fed a standard diet (SD; Purina Rodent Chow, Purina Mexico: protein 28.5%, lipids 13.5%, satured fats 1.5%, monounsatured fat 1.6%, and carbohydrates 58%) and the second one consisted of five rats fed a high‐fat diet (HFD; Purina Chow, Purina Mexico: protein 17.1% and lipids 47.5%, and carbohydrates 35.6%), whereas the extract‐treated groups consisted of five rats fed a HFD supplemented with nonirradiated strawberry extract 0.2% (HFD‐NSE) and five rats fed a HFD supplemented with UV‐irradiated strawberry extract 0.2% (HFD‐UViSE). The SD and HFD groups were fed during 20 weeks; whereas the HFD‐NSE and HFD‐UViSE groups were first fed a HFD during 12 weeks, then these groups were fed a HFD supplemented with NSE and UViSE, respectively, for 8 weeks. All the groups had access to water and food ad libitum. Food intake and body weight were recorded during the 20 weeks.

### 2.7 Anthropometric determinations

The abdominal circumference (AC) (immediately anterior to the forefoot), thoracic circumference (immediately behind the foreleg), body length (nose‐to‐anus or nose–anus length) were determined in all rats at the end of treatment. The body mass index (BMI) was calculated as body weight (g) divided by height squared (cm^2^). The measurements were made in anaesthetized rats (0.1 ml intraperitoneally of 1% sodium barbiturate).

### 2.8 Collection of blood and tissue samples

At the end of the 20 weeks treatment, the rats were sacrificed by cervical dislocation. Immediately, 3 ml of blood was collected directly from the heart and placed into tubes without anticoagulant, after of 20 min, the tubes were centrifuged at 1500 g for 5 min, and serum were recovered and stored at −20°C.

### 2.9 Determination of glucose, cholesterol, and triglycerides levels

In serum, the glucose was determined using the glucose oxidase‐peroxidase method (Biosystems, Spain). Total cholesterol and triglycerides levels were measured using enzymatic methods (STANBIO Laboratory, Boerne, Texas, USA).

### 2.10 Measurement of lipid peroxidation and oxidized proteins

The reactive aldehyde levels were quantified with the thiobarbituric acid‐reactive substances (TBARS) assay as indicative of lipid peroxidation, and carbonyls levels were measured as indicative of oxidized protein levels, following the instructions previously described (Martínez‐Morúa et al., 2013).

### 2.11 Statistical analysis

The statistical analyses were performed with Statistics for Windows 8 (StatSoft, Inc.). Each variable was applied normality test: Kolmogorov**–**Smirnov. Normally distributed data were represented as mean and standard error of the mean (S.E.M.). The ANOVA and post hoc Tukey′s test was used to find the differences between groups. The significance level set at *p* < .05.

## 3 RESULTS

### 3.1 Production of strawberry extracts

To increase the content of polyphenols, flavonoids, anthocyanins, and the antioxidant capacity in the strawberries, these were irradiated with UV‐C. The Table 1 shows that the UV irradiation significantly increased the content of polyphenols, flavonoids, anthocyanins, fisetin, and pelargonidin in the strawberry extract (SE) compared with nonirradiated strawberry extract (NSE). Then, the antioxidant capacity of the nonirradiated strawberry extract (NSE) and UV‐irradiated strawberry extract (UViSE) were determined. We observed that the antioxidant capacity in vitro was significantly increased in the UViSE compared with the NSE (1492 vs. 1035.7 nmol/L of TE/100 g of dry weight, *p =* .001).

**Table 1:** ‐Antioxidants content in the strawberry extracts and diets

Antioxidant molecules	NSE (mg/100 g of fresh weight)	UViSE (mg/100 g of fresh weight)	HFD‐NSE (mg/100 g of food)	HFD‐UViSE (mg/100 g of food)
Phenolic compounds	1265	3475.6^b^	2.53	6.95
Flavonoids	11.8	28.1^b^	0.024	0.060
Anthocyanins	50.3	108.9^b^	0.100	0.220
Fisetin	1.21	1.32^a^	0.0024	0.0026
Pelargonidine	8.32	13.1^a^	0.020	0.0260

HFD, high‐fat diet; HFD‐NSE, HFD supplemented with nonirradiated strawberry extract 0.2%; HFD‐UViSE, HFD supplemented with UV‐irradiated strawberry extract 0.2%.

^a^Compared with NSE (*p* < .05).

^b^Compared with NSE (*p* < .01).

As UViSE had higher antioxidant capacity in vitro than the NSE, then we determined whether this increased antioxidant capacity of the UViSE is also observed in vivo.

### 3.2 Effects of strawberry extracts on anthropometric parameters in obese rats

**Figure 1:**
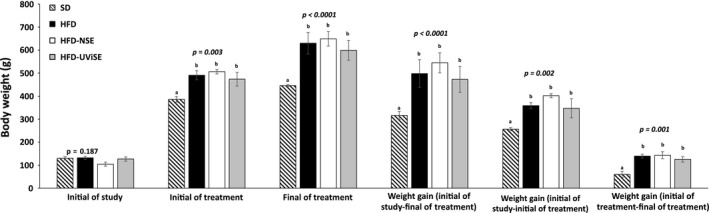
Effects of the HFD and strawberry extracts on body weight gain. SD, standard diet; HFD, high fat diet; HFD‐NSE, high‐fat diet + nonirradiated strawberry extract; HFD‐UViSE, high fat diet + irradiated strawberry extract. Data are given as the mean ± standard error of the mean (SEM)

It first was corroborated that the HFD to induce obesity in the rats. Thus, three groups were fed a HFD and other one was fed a SD. The rats were weighted at the beginning of the treatment and no significant differences were observed between the four groups (*p *=* *.187, Figure 1). At the 12 weeks of treatment, the HFD significantly increased body weight in the three groups of rats compared with the other one that was fed a SD, despite that, the food intake and consumption of energy were similar between the four groups (*p *= .003). Then the two control groups that were fed a HFD and SD continued with the same treatment until 20 weeks; whereas the others two groups that were fed a HFD received supplementation with the NSE and UViSE until completing a total of 20 weeks in order to determine the effects of these extracts on gain of body weight in the obese rats. At the end of the treatment (at the 20 weeks), the HFD, HFD‐NSE, and HFD‐UViSE groups significantly gained more body weight compared with the SD group (*p *= .0001), and no significant differences were observed between three groups of obese rats. Therefore, the 8 weeks of treatment with NSE and UViSE did have not any effects on the gain of body weight.

With respect to other anthropometric parameters, there were no significant differences in rat length between the four groups (Table 2). As expected, the SD group had significantly lower BMI compared with the HFD, HFD‐NSE, and HFD‐UViSE groups (*p* < .05). Similarly, the abdominal circumference (*p* < .05) and chest circumference (*p* < .01) were lower in SD group compared with the HFD, HFD‐NSE, and HFD‐UViSE groups, whereas these parameters were similar between the three groups of obese rats.

**Table 2:** Anthropometrical parameters

	SD (*n* = 5)	HFD (*n* = 5)	HFD‐NSE (*n* = 5)	HFD‐UViSE (*n* = 5)
BMI	0.723 ± 0.028	0.879 ± 0.031^a^	0.902 ± 0.037^a^	0.892 ± 0.0534^a^
Length	24.5 ± 0.612	26.8 ± 0.644	26.9 ± 0.828	26.0 ± 0.837
Abdominal circumference	18.54 ± 0.729	21.9 ± 0.510^b^	22.16 ± 0.44^b^	21.2 ± 0.644^a^
Thorax circumference	16.3 ± 0.464	18.7 ± 0.3^b^	19.0 ± 0.373^b^	18.8 ± 0.464^b^

SD, standard diet; HFD, high‐fat diet; HFD‐NSE, HFD supplemented with nonirradiated strawberry extract 0.2%; HFD‐UViSE, HFD supplemented with UV‐irradiated strawberry extract 0.2%; BMI, body mass index. Data are given as the mean ± SEM.

^a^Compared with SD (*p* < .05).

^b^Compared with SD (*p* < .01).

### 3.3 Effects of strawberry extracts on the food intake and consumption of energy in obese rats

**Figure 2:**
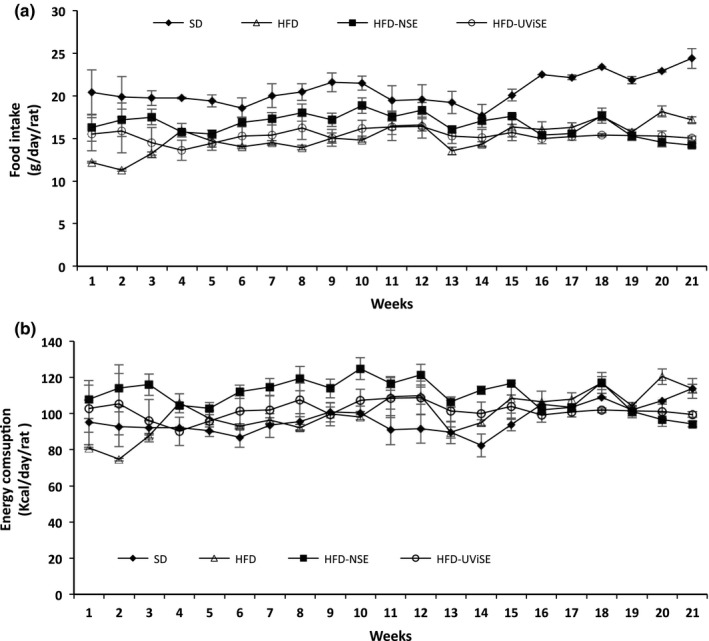
Effects of strawberry extracts on the food intake (a) and energy consumption (b). SD, standard diet; HFD, high fat diet; HFD‐NSE, high‐fat diet + nonirradiated strawberry extract; HFD‐UViSE, high fat diet + irradiated strawberry extract. Data are given as the mean ± standard error of the mean (SEM)

The effects of the NSE and UViSE on the food intake and consumption of energy in obese rats were determined. Both extracts did not have effect on the food intake neither on the consumption of energy compared with the control groups. Therefore, the food intake (in grams) and consumption of energy (kcal) were similar between the four groups (Figure 2a and b respectively).

Based on the daily mean amount of food consumed by the groups that received NSE and UViSE (16.65 ± 0.69 and 15.34 ± 1.02 g, respectively), and considering that the extracts were added to 0.2%, the consumption of antioxidants per day per rat was calculated. Thus, the antioxidants consumption by the NSE and UViSE groups was as follows: polyphenols (0.4213 and 1.0658 mg), flavonoids (0.0040 and 0.0092 mg), anthocyanins (0.0167 and 0.0337 mg), fisetin (0.0004 and 0.0004 mg), and pelargonidin (0.0033 and 0.0040 mg) respectively. Therefore, the HFD‐UViSE group consumed twice as much polyphenols, flavonoids, and anthocyanins than the HFD‐NSE group; whereas the consumption of fisetin and pelargonidin was similar between the two groups.

### 3.4 Effects of strawberry extracts on biochemical parameters in obese rats

Table 3 shows that the glucose levels were significantly lower (*p *< .05) in the SD‐fed rats than the HFD‐groups, and the SE did have not any effect on these levels. HFD increased nonsignificantly the triglycerides levels in the three groups that received HFD compared with the SD group. Moreover, the NSE extract decreased cholesterol levels in obese rats than the HFD group but it was not statistically different, whereas the UViSE had not any effects on cholesterol levels; HFD increased significantly HDL levels compared with other groups (*p* < .05). Finally, the NSE and UViSE did not change VLDL levels on the groups of obese rats.

**Table 3:** Biochemical parameters of rats

	SD (*n* = 5)	HFD (*n* = 5)	HFD‐NSE (*n* = 5)	HFD‐UViSE (*n* = 5)
Glucose	109.38 ± 12.56^a^	137.56 ± 5.82	162.02 ± 10.33^b^	148.90 ± 11.19
Triglycerides	81.54 ± 7.14	109.34 ± 13.52	96.82 ± 2.44	163.52 ± 41.83
Cholesterol	89.32 ± 7.66^a^	117.16 ± 3.08^b^	97.84 ± 5.37	112.22 ± 10.74
HDL	76.84 ± 2.59^a^	89.22B ± 4.3^b^	74.52 ± 1.71^a^	75.88 ± 2.93^a^
VLDL	16.28 ± 1.43	21.82 ± 2.70	19.34 ± 0.49	32.66 ± 8.37

SD, standard diet; HFD, high‐fat diet; HFD‐NSE, HFD supplemented with nonirradiated strawberry extract 0.2%; HFD‐UViSE, HFD supplemented with UV‐irradiated strawberry extract 0.2%; HDL, high density lipoprotein; VLDL, very low density lipoprotein. Data are given as the mean ± SEM.

^a^ vs. ^b^ (*p* < .05).

### 3.5 Effect of strawberry extracts on the brain oxidative damage

**Figure 3:**
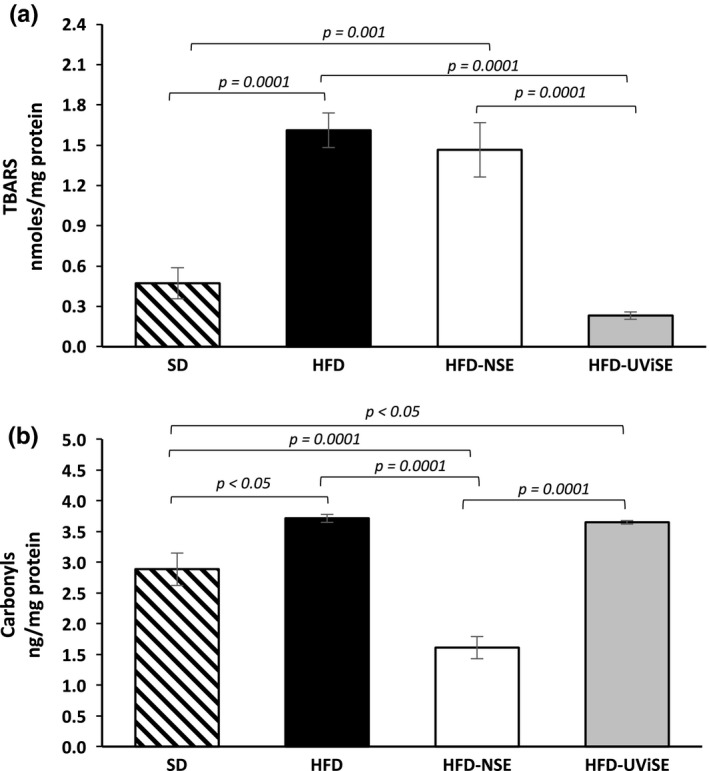
Effects of strawberry extracts on the oxidative damage in the cerebellum. SD, standard diet; HFD, high fat diet; HFD‐NSE, high‐fat diet + nonirradiated strawberry extract; HFD‐UViSE, high fat diet + irradiated strawberry extract. Data are given as the mean ± standard error of the mean (SEM)

First, the effects of HFD on the cerebellum lipid peroxidation were determined. Figure 3a shows that TBARS levels were significantly higher in HFD group compared with the SD (*p* = .0001). Interestingly, the UViSE significantly reduced the lipid peroxidation, HFD‐UViSE grup, compared with the HFD group (*p* = .0001), this reduction was as low as the oxidation levels in the SD group. The NSE had no significant effect on lipid peroxidation as compared with the HFD group. Figure 3b shows that the carbonyls levels were higher in the HFD group compared with the SD group (*p < *.05), whereas the NSE significantly reduced (*p =* .0001) these levels compared with the SD, HFD and HFD‐UViSE groups. The UViSE did not have any effect on the carbonyls levels, having the HFD‐UViSE group higher carbonyls levels than the SD group (*p *< .05).

**Figure 4:**
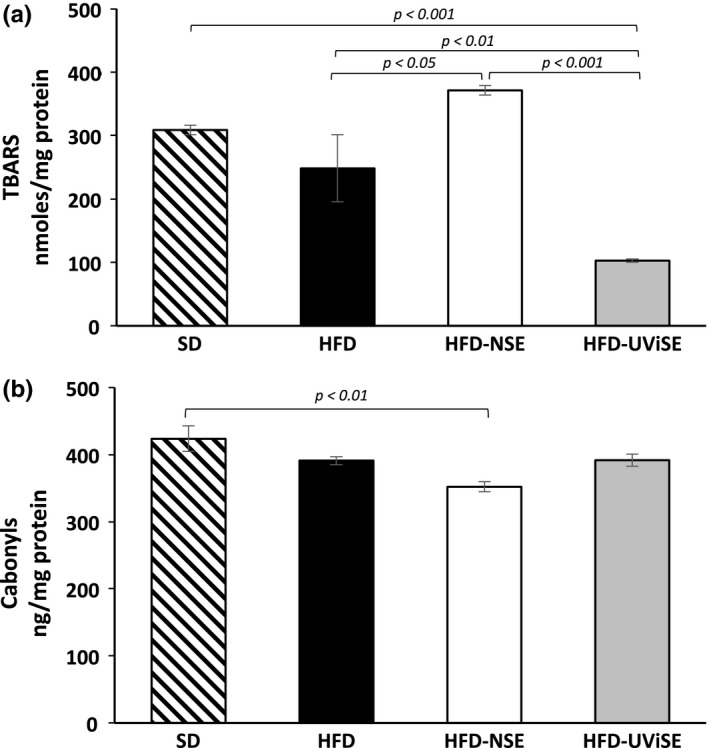
Effects of strawberry extracts on the oxidative damage in hippocampus. SD, standard diet; HFD, high fat diet; HFD‐NSE, high‐fat diet + nonirradiated strawberry extract; HFD‐UViSE, high fat diet + irradiated strawberry extract. Data are given as the mean ± standard error of the mean (SEM)

Second, oxidative damage on hippocampus was determined. The HFD and NSE had no significant effect on the lipid peroxidation levels, and contrary the HFD‐NSE group had higher lipid peroxidation levels compared with the HFD group (*p* < .05, Figure 4a). Interestingly, the UViSE significantly reduced lipid peroxidation levels compared with the HFD and HFD‐NSE groups (*p *< .01 and *p *< .001, respectively), being these levels lower than the SD group (*p* < .001). With respect to carbonyls levels (Figure 4b), these levels were similar among the groups, only the groups HFD‐NSE had lower carbonyls levels compared with SD group (*p *< .01).

### 3.6 Effect of strawberry extracts on the pancreas oxidative damage

**Figure 5:**
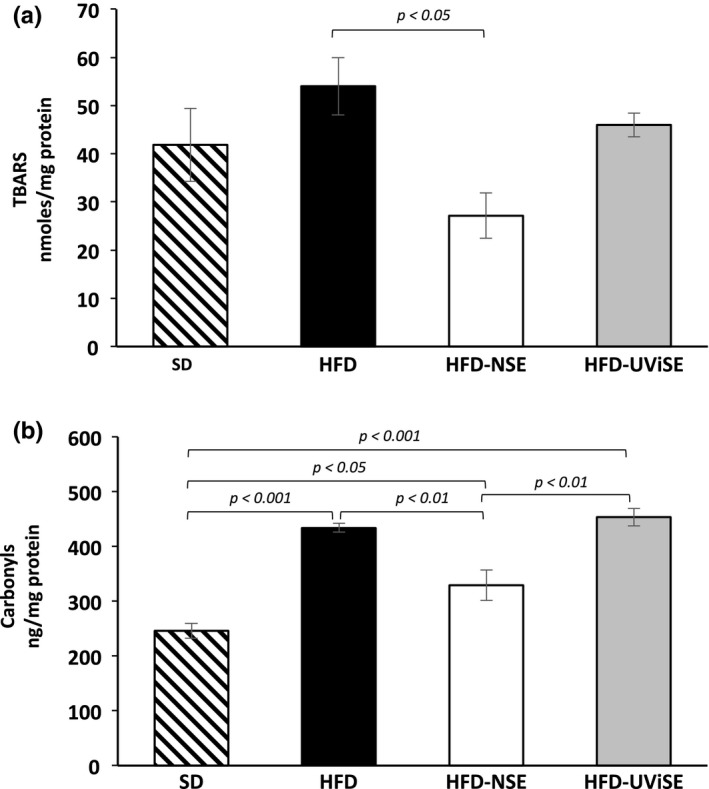
Effect of strawberry extracts on the pancreas oxidative damage. SD, standard diet; HFD, high fat diet; HFD‐NSE, high‐fat diet + nonirradiated strawberry extract; HFD‐UViSE, high fat diet + irradiated strawberry extract. Data are given as the mean ± standard error of the mean (SEM)

It was observed that the HFD weakly increased the lipid peroxidation levels compared with the SD group, whereas the NSE significantly decreased these levels in obese rats compared with HFD groups (*p *< .05), but the UViSE had no significant effect (Figure 5a). The effects were more clearly observed in carbonyls, where HFD significantly increased carbonyls levels than the SD group (*p *< .001), whereas the NSE significantly reduced carbonyls levels in the obese rats compared with nontreated obese rats (*p *< .01) (Figure 5b). Again, the UViSE had no significant effect on carbonyls levels in pancreas.

### 3.7 Effect of strawberry extracts on the liver oxidative damage

**Figure 6:**
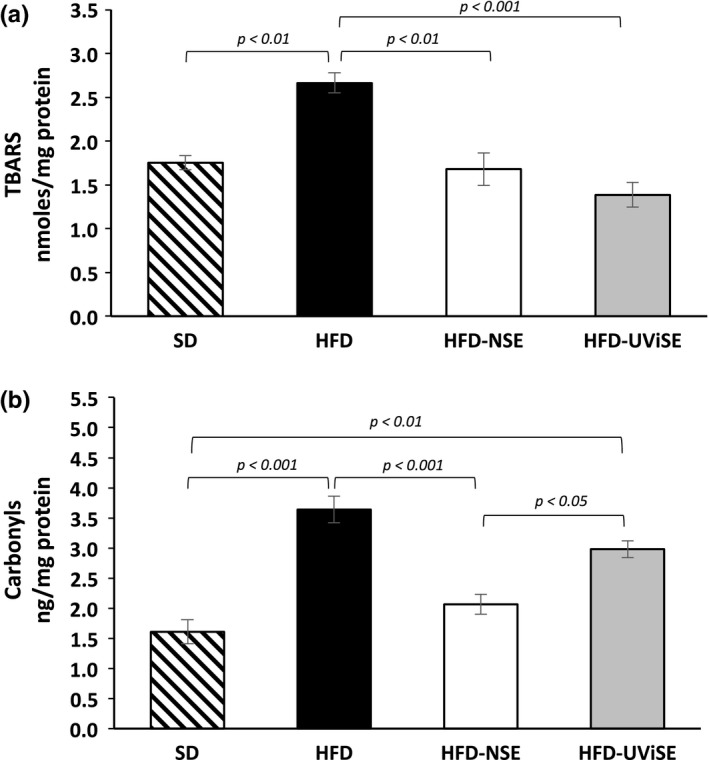
Effect of strawberry extracts on the liver oxidative damage. SD, standard diet; HFD, high fat diet; HFD‐NSE, high‐fat diet + nonirradiated strawberry extract; HFD‐UViSE, high fat diet + irradiated strawberry extract. Data are given as the mean ± standard error of the mean (SEM)

As shown in Figure 6a, the lipid peroxidation levels were higher in liver of obese rats compared with the SD group (*p *< .01); interestingly, both NSE and UViSE significantly decreased (*p *< .01 and *p *< .001, respectively) the lipid peroxidation levels as compared with the HFD group. With respect to carbonyls levels (Figure 6b), clearly the HFD significantly increased these levels than the SD (*p *< .001), but only the NSE significantly reduced the carbonyls levels as compared with the HFD group (*p *< .001), being significantly different the carbonyls levels between NSE and UViSE groups (*p *< .05).

### 3.8 Effect of strawberry extracts on the kidney oxidative damage

**Figure 7:**
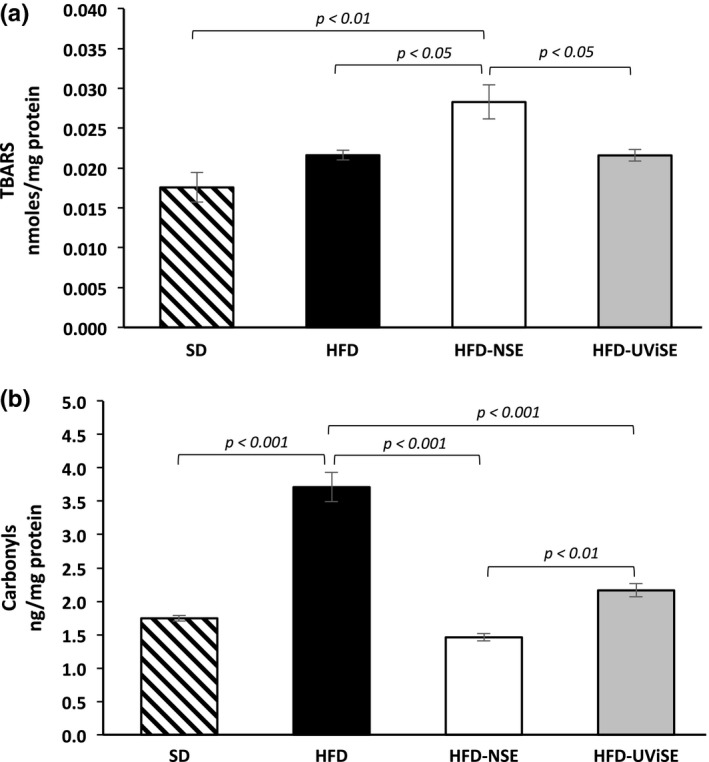
Effect of strawberry extracts on the kidney oxidative damage. SD, standard diet; HFD, high fat diet; HFD‐NSE, high‐fat diet + nonirradiated strawberry extract; HFD‐UViSE, high fat diet +  irradiated strawberry extract. Data are given as the mean ± standard error of the mean (SEM)

As shown in Figure 7a, the HFD did not have any effect on the lipid oxidation levels. Contrary with expectation, the NSE significantly increased the lipid peroxidation levels in obese rats as compared with the SD and HFD groups (*p *< .01 and *p *< .05, respectively), whereas the UViSE did not have any effect on the lipid peroxidation as compared with the HFD group. With respect to the carbonyls levels (Figure 7b), the HFD significantly increased these levels as compared with the SD group (*p < *.001); whereas both NE and UViSE significantly reduced carbonyls levels as compared with HFD group (*p *< .001). Importantly, the NSE was more effective to reduce the carbonyls levels compared with the UViSE (*p *< .01).

### 3.9 Effect of strawberry extracts on the heart oxidative damage

**Figure 8:**
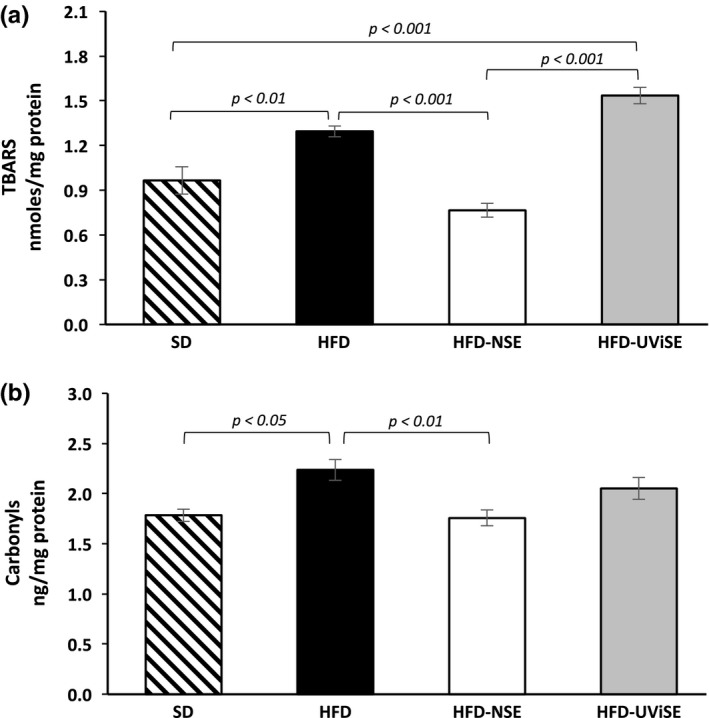
‐ Effect of strawberry extracts on the heart oxidative damage. SD, standard diet; HFD, high fat diet; HFD‐NSE, high‐fat diet + nonirradiated strawberry extract; HFD‐UViSE, high fat diet + irradiated strawberry extract. Data are given as the mean ± standard error of the mean (SEM)

In heart, the HFD increased lipid peroxidation levels (Figure 8a), compared with the SD (*p *< .01), whereas the NSE significantly reduced these levels in obese rats as compared with the HFD group (*p *< .001). Unfortunately, the UViSE significantly increased lipid peroxidation compared with the SD and HFD‐NSE groups (*p *< .001). The Figure 8a shows that HFD also increased carbonyls levels compared with the SD (*p *< .05), and the NSE importantly reduced these levels in obese rats compared with the nontreated obese rats (*p *< .01). The UViSE weakly decreased carbonyls levels in obese rats as compared with the HFD group, and this weak reduction was sufficient to reverse the significant increase produced by the HFD (Figure 8b).

## 4 DISCUSSION

It is well‐known that the strawberries have a high content of antioxidants (anthocyanins, tannins, catechin, quercetin, kaempferol, gallic acid derivatives, vitamins C, E, and carotenoids) (Kårlund et al., 2014). Moreover, it has been suggested that UV‐irradiation increase the content of these antioxidant in the fruits (Alothman et al., 2009; Perkins, Collins, Perkins‐veazie, Collins, & Howard, 2008; Younis et al., 2010). Therefore, in the present study, we first irradiated strawberry slices with UV‐C; then, strawberry extract was prepared as described above. Second, we determined the antioxidant capacity of this extract. Our results demonstrated that the irradiation with UV‐C significantly increased flavonoids, phenols, and anthocyanins content in the strawberry extract than the nonirradiated strawberry extract. Irradiation with UV‐C also increased antioxidant capacity of the strawberry extract as was determined using the TEAC assay with tube reactions. This increased antioxidant capacity is probably the result of the successful induction of the antioxidant defense of the strawberry, which increases the production of secondary metabolites to decrease the oxidative stress caused by the UV light. It was reported that UV‐C increases the total anthocyanin and total phenolic content of strawberry. Moreover, UV‐C enhanced the activities of antioxidant enzymes including glutathione peroxidase (GSH‐POD), glutathione reductase (GR), SOD, ascorbate peroxidase (AsAPOD), guaiacol peroxidase (G‐POD), monodehydroascorbate reductase (MDAR), and dehydroascorbate reductase (DHAR) (Erkan, Wang, & Wang, 2008). Others authors reported that the UV‐C treatment increased the total phenol, and flavonoid of fresh‐cut honey pineapple, banana, and guava (Alothman et al., 2009; González‐Aguilar, Zavaleta‐Gatica, & Tiznado‐Hernández, 2007).

Once we demonstrated that UV‐C radiation increases the antioxidant capacity in vitro; then we determined whether this antioxidant capacity occurs in vivo; for which, we feed to rats with HFD. As predicted, we found that the HFD increased the body weight and the blood levels of glucose and cholesterol in rats. Moreover, HFD also increased lipid and protein oxidation in the cerebellum and in peripheral organs of rats. Ours results are consistent with previous reports where it was observed that HFD increased oxidative stress in brain (Freeman et al., 2014; Younis et al., 2010) and peripheral organs (Charradi, Elkahoui, Limam, & Aouani, 2013; Martínez‐Morúa et al., 2013).

HFD‐induced obese rats were treated with the NSE and UViSE, obtaining the follow results: First, our results show that although the rats had access to food ad libitum, the SD group ate more food compared with the three groups of HFD, but the consumption of energy was very similar among the four groups. Spite that, the HFD induced efficiently obesity in rats; it is likely that the high content of lard fat and carbohydrates was responsible to induce obesity in ours rats. It is well‐known that saturated fat and complex carbohydrates are more effective to induce obesity (Ble‐Castillo et al., 2012; Brown et al., 2010), and it also has been suggested that the protein diets are more effective for weight loss by providing the twin benefits of improving satiety and decreasing fat mass (Pesta & Samuel, 2014). Thus, the body weight gain observed in the present study is consistent with studies in animal models, suggesting that exposure to high concentrations of carbohydrates or HFD contribute to the development of overweight and obesity (Williams, Seki, Vuguin, & Charron, 2014).

Second, our results show that the NSE and UViSE did not have any significant effect on the food intake, consumption of energy and gain of body weight. In contrast, it was reported that raspberry seed oil decreases of liver fat content and atherogenic index in healthy rats and rats with low‐grade systemic inflammation (Jurgoński, Fotschki, & Juśkiewicz, 2015), whereas in alloxan‐induced diabetic rats the strawberry extract was effective to restore the body weight loss and hyperglycemia (Abdulazeez & Ponnusamy, 2016). Likely, these differences between our data and these reported by others are due others used raspberry seed oil, whereas we used strawberry extracts. Moreover, we used obese rats and others used diabetic rats.

Third, our results show that the NSE and UViSE did not have any important effect on cholesterol, triglycerides, HDL, VLDL, and glucose levels. It is supported by a previous study where strawberry seed oils had no significant effect on cholesterol, triglycerides, HDL, and LDL levels in rats (Pieszka, Tombarkiewicz, Roman, Migdał, & Niedziółka, 2013). In contrast with our data, others studies demonstrated that the strawberry improves the hyperglycaemia and blood lipid profile in human. For instant, 10 g of lyophilized strawberry per day for 6 weeks reduced triglycerides and LDL levels in serum of men and women (Burton‐Freeman, Linares, Hyson, & Kappagoda, 2010), and in obese human subjects a diet with lyophilized strawberry during 3 weeks reduced plasma concentration of cholesterol and LDL (Zunino et al., 2012). However, 10 g of lyophilized strawberry per day for 6 weeks did not have any effect on blood glucose levels in overweight men and women (Ellis, Edirisinghe, Kappagoda, & Burton‐Freeman, 2011). We suggested that the strawberry had no effect on the blood lipid profile and glucose levels when the strawberry organic phase is administrated as was performed in our study and in the study performed by Pieszka (2013). Thus, the strawberry has benefic effect on the blood lipid profile and glucose levels just when it is administrated with her fiber content as was performed in human (Burton‐Freeman et al., 2010; Ellis et al., 2011; Zunino et al., 2012).

Fourth, with respect to antioxidant capacity of the NSE and UViSE, as we mentioned above, the UViSE had higher antioxidant content and antioxidant capacity in vitro than the NSE. We also observed this effect in brain (cerebellum and hippocampus) where the UViSE had better effect on lipid peroxidation than the NSE; whereas the NSE had better effect on protein oxidation only in cerebellum than the UViSE. However, in peripheral tissues, the UViSE's antioxidant capacity was no better than the NSE; therefore, both extracts had similar antioxidant capacity, sometime the NSE was better than the UViSE, and sometime the UViSE was better than the NSE. The beneficial effects of our extracts can be due to the strawberry flavonoids, which have been shown that play a key role in human health. The most abundant antioxidants in the strawberry are caffeic acid, ellagic acid, and certain flavonoids including anthocyanins, tannins, catechin, quercetin, kaempferol, gallic acid derivatives, vitamins C, E, and carotenoids (Kårlund et al., 2014). In addition, flavonoids of fruits and vegetables have been correlated with a reduced incidence of cardiovascular deceases and control glucose levels (Giampieri, Alvarez‐Suarez, & Battino, 2014; Giampieri et al., 2014; Tulipani, Mezzetti, & Battino, 2009).

We found that in pancreas the NSE extract decreases the lipid oxidation and carbonyl levels compared to HFD group. These findings are very important because in pancreas, a study found that long‐term to high fat promotes oxidative stress followed by detrimental to Beta cells, producing activation of apoptotic ways (Zhao et al., 2015). Moreover, the liver plays an important role in complex metabolism like glycogen storage, lipid and protein synthesis, and detoxification (Hamed et al., 2016), for this reason, impairment liver function can lead to increase oxidative stress (Hamed et al., 2016). In our study, HFD increased the TBARS and carbonyls levels, whereas the NSE and UViSE extracts reduced these markers of oxidative damage; thus, ours extracts protect to the liver against oxidative damage, but unfortunately the effect of UViSE on protein oxidation was not significant. Likewise the strawberry has been used as hepatoprotective in liver injury by tetrachloride in rats, which is due to his anti‐apoptotic and antioxidant properties (Hamed et al., 2016).

The present results show that HFD increases the protein oxidation and that the NSE and UViSE extracts reduce this oxidation in kidney, but these extracts do not reduce lipid peroxidation. Thus, our data are very important because it was described that HFD induces proteinuria, glomerular hypertrophy, glomerular fibrosis, tubular injury, and oxidative stress (Odermatt, 2011). Moreover, it has been demonstrated that hyperlipidemia produce oxidative stress which is implicated on the development of glomerular injury (Scheuer et al., 2000). Contrary with kidney, we found that in the heart, the HFD and UViSE increased lipid peroxidation than the SD. Importantly, the NSE reduced the lipid and protein oxidation compared with the HFD, but the UViSE had no effect on protein oxidation. Others have described that berries have polyphenols like anthocyanins and micronutrients, and that these components are associated with an reduction in cardiovascular risk in humans and in experimental animals models (Basu et al., 2010). These discrepancies may be explained by the sample size, dose and type of strawberry (extracts or purified components).

Finally, our results show that UV‐irradiation increased the antioxidants content and antioxidant capacity of the strawberry in vitro, but in vivo, it does not improve the oxidative stress in all organs. This increased antioxidant capacity was better in brain than peripheral tissues, likely by increasing the content of antioxidants that crossing the blood‐brain barrier such as the quercetin. It is very important because the UV‐irradiated‐strawberry supplementation may reduce the brain oxidative stress and prevent the development of neurodegenerative diseases.

## 5 CONCLUSION

UV‐irradiation increases antioxidant capacity of strawberry, but this could be more effective in brain than peripheral tissues. Thus, the role of strawberry in modulating the antioxidant and inflammatory biomarkers needs to be more rigorously examined in future trials with different organs.

## CONFLICT OF INTEREST

The authors declare that they have no conflict of interest.
